# Information, assessment, or decision: a driving simulator study on the effect of real-time feedback based on information-processing stages

**DOI:** 10.1080/00140139.2025.2477624

**Published:** 2025-07-14

**Authors:** Angèle Picco, Arjan Stuiver, Joost de Winter, Dick de Waard

**Affiliations:** a Faculty of Behavioural and Social Sciences, University of Groningen, Groningen, The Netherlands; b Faculty of Mechanical, Maritime and Materials Engineering, Delft University of Technology, Delft, The Netherlands

**Keywords:** Real-time feedback, information processing stages, driving, speeding, time-headway

## Abstract

This driving simulator study, which focused on supporting drivers through feedback rather than automating the driving task, examined the effect of real-time feedback based on different stages of information processing on driving behaviour. The stages investigated included providing information alone, assessment of that information, and a decision based on that assessment, following Parasuraman, Sheridan, and Wickens’s (2000) model of information-processing automation. The acceptability and effectiveness of the different stages of feedback were assessed on two key driving behaviours: speed and distance from the vehicle ahead. The results indicated that feedback had a limited effect on driving behaviour. However, the stage of information processing in the feedback did affect a number of outcomes, with decision-oriented feedback leading to improved behaviours but less favourable attitudinal results. Future safety interventions should consider altering risk perception and beliefs, or providing external motivation for behavioural change.

## Practitioner summary

This simulator study investigated the effects of real-time feedback on driving behaviour. The feedback concerned different stages of processing: information, analysis, and decision. Results showed that decision feedback was most effective, although effects were small. This suggests that other factors, such as incentives, may be more important than providing feedback.

## 1. Introduction

Driving is a common everyday activity. In the Netherlands, for example, over 80% of people aged 17 and above hold a driving licence, and an average of 14 km is driven per person daily (CBS 2019, 2022a). While often seen as mundane, driving is far from infallible, with 522 fatalities occurring on Dutch roads in 2021 (CBS 2022b). Identifying and understanding the factors behind driving failures could enable the development of effective interventions and support strategies for drivers that may, if effective, enhance road safety.

Driving is a complex task that can be conceptually simplified as an information-processing activity: the driver processes information from the environment to respond to it. The information processing system is historically described as a four-stage process: (1) sensory processing, (2) information analysis, (3) decision selection, and (4) action implementation (Smith 1968; Welford 1960; Wickens 1974; Wickens and Carswell 2021). This process conceptually captures the fundamental steps involved in driving: drivers extract information, primarily through visual and motion cues (Mcruer and Weir 1969), but also through auditory and potentially even olfactory stimuli. They then analyse the information to assess the situation, based on their experience and their knowledge, as well as on the time available. Based on their assessment, and complex mechanisms involving motivational factors as well as experience, drivers make decisions on a response they deem appropriate. Note that these information-processing stages do not necessarily occur at a high level of consciousness. Especially for experienced drivers, decision-making and execution processes are largely automated, i.e. System 1 behaviour according to Kahneman (2011) or skill-based behaviour according to Rasmussen (1983). Nonetheless, whether conscious or more automatic, the information-processing stages remain the same: information acquisition, analysis, and decision-making. Drivers finally put into action this decision, which results in a change in the environment, calling for a new loop of this information-processing task.

While theoretically straightforward, the information-processing loop contains several opportunities for failure. Classifications of human errors in driving are numerous (see Stanton and Salmon (2009, for an overview), but the classification proposed by Treat et al. (1979) aligns well with Parasuraman, Sheridan, and Wickens (2000)’s information processing model. Treat et al. distinguished between recognition, decision, and performance errors. Recognition errors refer to both identifying and assessing situations incorrectly, due, for example, to inattention or distraction (Singh 2018). Decision errors refer to flawed decision-making and can occur either for motivational reasons (i.e. deciding to take risks, such as driving faster than recommended) or capability reasons (i.e. not knowing the appropriate reaction for the situation). Recognition and decision errors account for 41% and 33% respectively of crashes for which the critical reason is the driver (Singh 2018). Finally, performance errors entail poor execution of the intended action and comprise about 11% of crashes (Singh 2018). While human error alone does not explain all crashes and risky behaviours, and not all human errors are recognition, decision, or performance errors, the information processing involved in driving presents multiple opportunities for failure.

In their work, Parasuraman, Sheridan, and Wickens (2000) introduced a similar four-stage information-processing model to that of Wickens (Wickens 1974; Wickens and Carswell 2021), but applied it to automation systems. They described that automated systems, like humans, acquire information, analyse this information, make decisions, and execute actions. Parasuraman et al. also described that automation can occur at different levels, where the human remains involved (low level of automation) or is supplanted (high level of automation). This is in line with more recent works by Navarro (2017) and Banks, Stanton, and Harvey (2014), who explained that automation systems in driving can either ‘support’ or ‘replace’ human driving activity. In the current study, the choice is made to support drivers and help them perform better, not replace them. Instead of focusing on automating the driving task execution itself, this study aims to automate certain aspects of the driving activity and present the results to the driver. In this regard, the first three stages of the information processing system can be automated, with the results provided to the driver as support. Since the last stage, ‘action implementation’, would replace rather than support the driver, it is not included. Automating the first three stages of the information processing system can be achieved by providing feedback, as demonstrated by Eriksson et al. (2019). In that study, drivers received different stages of processed traffic-related information in the form of feedback to help improve their take-over performance from automated driving.

The use of feedback to improve driving performance is not new: about two decades of research have now been dedicated to investigating the effects of feedback on driving behaviour (e.g. Bell et al. 2017; De Waard, Van der Hulst, and Brookhuis 1999; Goodrich and Quigley 2004; Mase et al. 2020; Toledo and Lotan 2006; Toledo, Musicant, and Lotan 2008). Yet, most studies have examined how to provide feedback, focusing on timing (real-time vs. delayed), positioning (e.g. head-up vs. head-down), intensity, or modality (in-vehicle device, smartphone app, website, human coaching; or auditory, haptic, visual, etc.), but the stage of information processing in the feedback has not been extensively investigated experimentally. Previous studies show this question is relevant: an interview study on using data in driving exams found that examiners prefer having access to raw (least processed) data and not just processed data, as they want to remain in charge of assessing the information (Driessen et al. 2021). Taking for example the distance between the ego-vehicle and other road users, the examiners favoured having access to the numerical distance (the raw measurement) rather than just having access to an evaluation, such as whether the vehicle was driving too close or far enough. Similarly, in an acceptability study of monitoring and feedback devices, respondents also preferred more precise (less processed) information (Picco et al. 2023). While experts and non-experts seem to prefer less processed information, its effect on driving behaviour remains unknown, as preferences do not always indicate effectiveness. For example, Degirmenci and Breitner (2023) observed a trade-off between experiential outcomes (e.g. ease of use, enjoyment, and intention to use) and instrumental outcomes when providing gamified feedback, confirming that a preference for a type of feedback does not necessarily equate to its effectiveness.

This study, therefore, aims to investigate whether feedback based on different stages of processing of the same information produces different effects on behaviour. The stages are (1) information alone, (2) assessment of that information, and (3) a decision based on that assessment, in line with Parasuraman, Sheridan, and Wickens (2000). Better results when providing feedback in the first condition (Information) would indicate fallibility in drivers’ ability to acquire information, better results in the second condition (Assessment) would indicate fallibility in interpreting the information acquired, and better results in the last condition (Decision) would indicate fallibility in associating a certain assessment with the need to change behaviour. To test these hypothesised effects, feedback was provided on two relevant behaviours: (1) speed, a key factor in accident fatalities (Elvik 2005); and (2) distance from the vehicle ahead, another indicator of risky and aggressive driving (Kerwin and Bushman 2020; Lewis-Evans, De Waard, and Brookhuis 2010). If feedback leads to a behaviour change, it can indicate which stage of information processing is needed to support the driver. In contrast, if no effect is observed, reasons for risky behaviours could be of motivational origins rather than an issue of capabilities. In addition to these research questions, the acceptability of the different stages of feedback was assessed, as acceptability is a predictor of eventual use (Van Der Laan, Heino, and De Waard 1997), and therefore of the feedback’s effectiveness.

## 2. Methods

### 2.1. Participants

A total of 34 participants were recruited, with 29 completing the study after five dropped out due to simulator sickness. The sample consisted of 15 women and 14 men, aged 18 to 62 (*M* = 33.5, *SD* = 17.6). On average, participants had held their licences for 14.6 years (*SD* = 16.3) and had obtained them at an average age of 18.9 (*SD* = 2.4). The majority (*n* = 23) were Dutch nationals, followed by 3 Germans and 3 Eastern Europeans. Only 10 participants mainly drove private cars, 17 biked, 1 walked, and 1 used public transit. Nine drove 1–1,000 km/year; 7 drove 1,001–5,000 km; 5 drove 5,001–10,000 km; 2 drove 10,001–20,000 km; 4 drove 20,001–30,000 km; 1 drove 30,001–50,000 km; and 1 drove 50,001–100,000 km.

As a means to describe the sample, the participants were asked to self-assess their driving skills and to indicate their opinions about driving on Likert scale items ranging from 1 (Strongly disagree) to 7 (Strongly agree). These results are presented in Table 1. Participants were, for example, enthusiastic about driving (*M* = 6.0), would rate themselves as good drivers (*M* = 5.4), and slightly better than the average driver (*M* = 4.3), yet believed there was still room for improvement in their driving (*M* = 5.3). They were also rather positive towards technology (*M* = 4.6), assessed with a shortened version of the Affinity for Technology Interaction (ATI) scale (Franke, Attig, and Wessel 2019).

**Table 1.** Mean and standard deviations to Likert scale items.

Likert scale items (1-Strongly disagree to 7-Strongly agree)	Mean	SD
*Before the experiment*		
I enjoy driving a car	6.0	0.9
I am a good driver	5.4	0.8
I am better than the average driver	4.3	1.3
When it comes to my driving ability, there is still room for improvement	5.3	1.1
If you are a good driver it is acceptable to drive a little faster	2.8	1.4
It is acceptable to drive when traffic lights change from green to yellow	4.6	1.4
Technology acceptance score (range 1–7)	4.6	1.1
*After the experiment*		
I sometimes have difficulties assessing the speed I am driving at	3.3	1.8
I sometimes have difficulties assessing the distance between my car and the car in front of me	3.6	1.7

Participants also reported no apparent difficulties assessing either their speed (*M* = 3.3) or the distance between their car and the car in front of them (*M* = 3.6). These two items were only asked after the experiment to avoid influencing the outcome of the study. It was reasoned that having participants reflect on their behaviour before the experiment could have influenced their driving and their reliance on the feedback.

Participants were recruited through convenience sampling, social media, and flyers (combined *n* = 13), and from a first-year students’ subject pool (*n* = 16). Inclusion criteria were: being at least 18 years old, proficient in Dutch or English, and holding a valid driving licence. Participants from the student pool received course credit, while three participants from the other group were randomly selected to receive 25 euros each as an incentive and token of appreciation.

The study was approved by the Ethics Committee of the Faculty of Behavioural and Social Sciences of the University of Groningen (research code: PSY-2223-S-0011).

### 2.2. Materials

#### 2.2.1. Simulator

The driving part of the experiment took place in a Jentig50 driving simulator (ST Software). The setup consisted of five 60-inch diagonal LED screens and an open cabin mock-up placed on a moving base (CKAS Mechatronics), including a driving seat, steering wheel, gearbox and pedals. The simulator was used in an automatic gearbox mode. The graphical interface and routes were designed using StRoadDesign and the scenarios were programmed using StScenario, two packages of the ST Software.

#### 2.2.2. Route

A route of approximately 11 km was created for this experiment. It included provincial roads with speed limits of 80 km/h or 60 km/h in rural areas and 50 km/h in urban areas, and a four-lane motorway with a 100 km/h limit. The route was designed to mimic realistic conditions by incorporating various types of roads and surroundings, such as urban and rural areas, with different lane widths. The route was also populated with traffic: oncoming traffic was created pseudo-randomly during the whole experiment and specifically at intersections. In some segments, there was also a car driving in front of the ego-vehicle, to perform a car-following task, thus enabling determination of time headway (THW). Traffic was programmed to drive adhering to speed limits for the whole drive, except on the motorway where traffic was less homogeneous, with some cars driving slower and others faster than the speed limit. On average, the drive lasted about 10 minutes, and was, in total, completed four times by the participants during the experiment. The procedure is detailed in Section 2.3.

**Figure 1.**
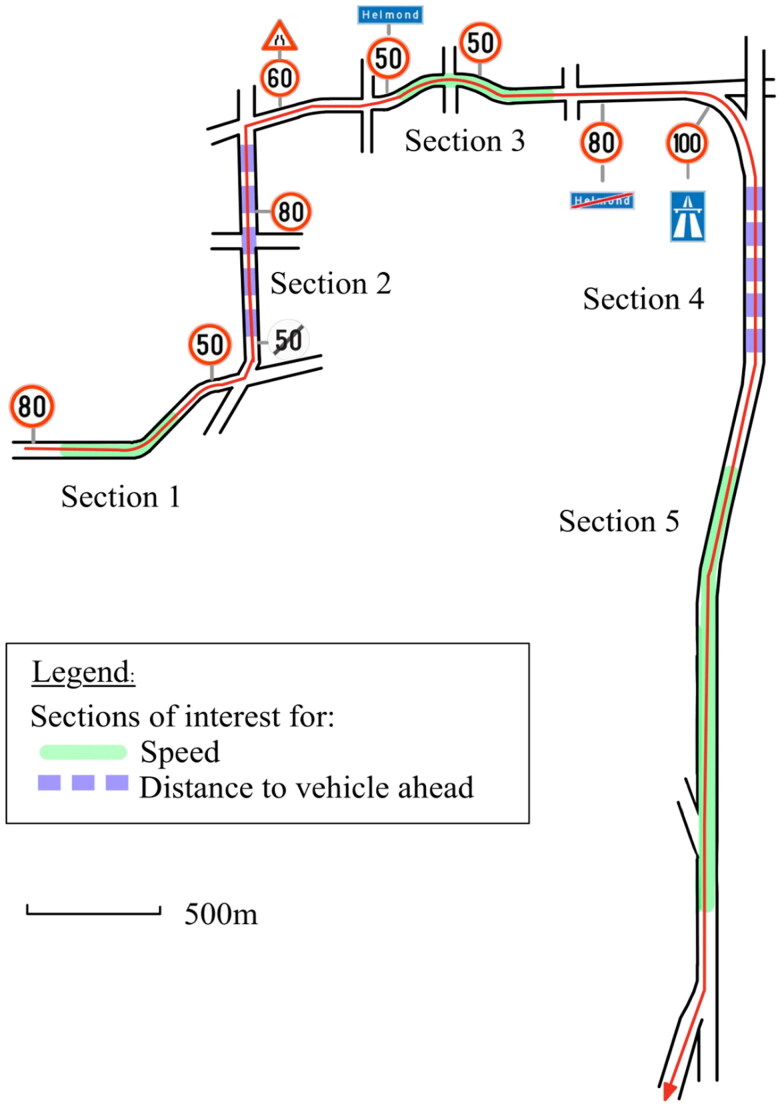
Map of the experiment’s route.

This route is illustrated in Figure 1. The figure also depicts sections of interest: while the participants’ behaviours were recorded throughout the whole drive, particular sections were identified and aimed at analysing behaviours depending on the context, as described in Section 2.4.1.

#### 2.2.3. Conditions

**Figure 2.**
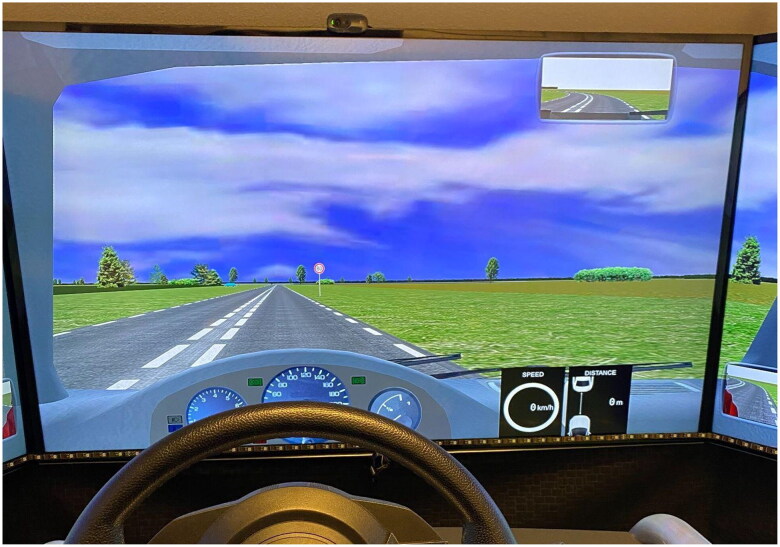
Simulator set-up depicting the ‘feedback zone’ on the screen.

A within-subjects design with three experimental conditions, Information, Assessment and Decision, and one control condition was used. In all experimental conditions, feedback was provided, differing in the stage of information processed. In the control condition, no feedback was given. The feedback pertained to the participants’ speed and the distance between their vehicle and the vehicle in front of them. For this purpose, a ‘feedback area’ was positioned on the lower right-hand section of the dashboard, at the bottom part of the central screen, showing two black squares where information could be displayed (as displayed in Figure 2); one for each type of behaviour of interest.

In the Information condition, the feedback presented a numerical value, specifically, the current speed in km/h and the distance to the lead vehicle in metres. In the Assessment condition, the feedback provided an assessment of these behaviours, indicating whether they were violating traffic rules or not by either a thumbs up or thumbs down icon. And, in the Decision condition, the feedback suggested a behaviour to adopt, by either showing the text ‘no change needed’, ‘slow down’ or ‘increase distance’. The specific illustrations and choice of words were chosen after collecting the opinions of 28 people in a short questionnaire study, to assess the understandability of the feedback and the participants’ preferences. These are presented in Table 2.

**Table 2.** Feedback images.

	Condition		
	Information	Assessment	Decision
Feedback on speed	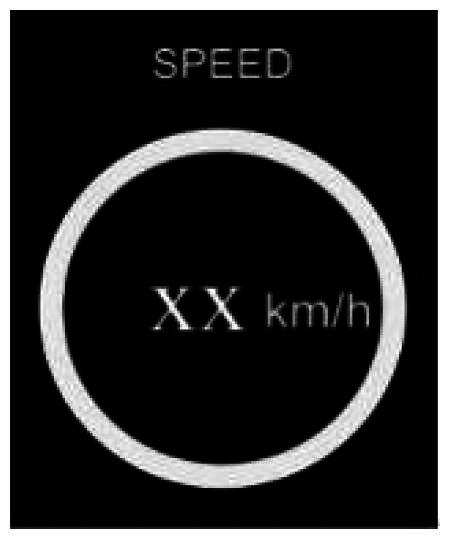	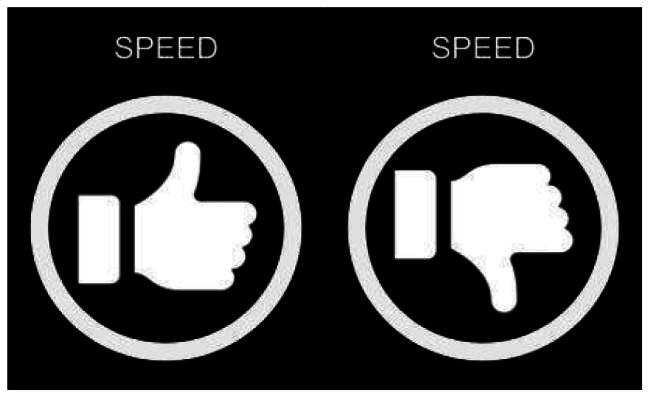	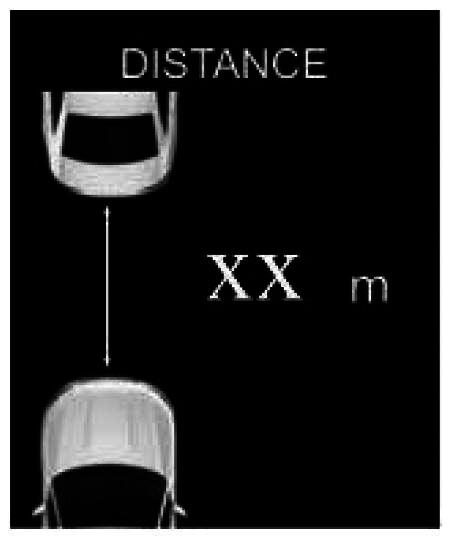
Feedback on distance	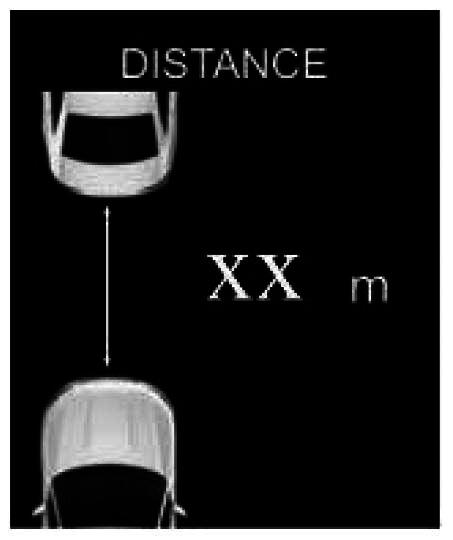	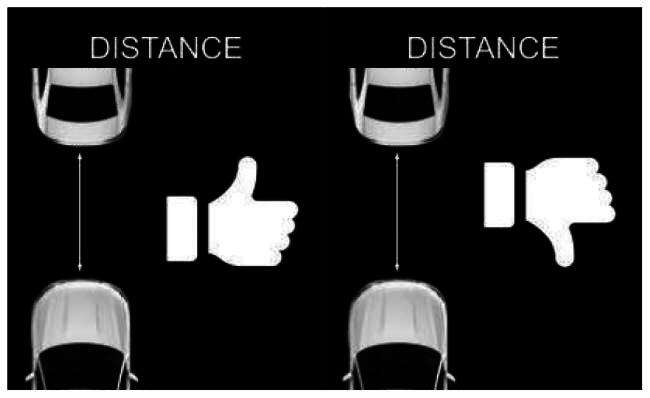	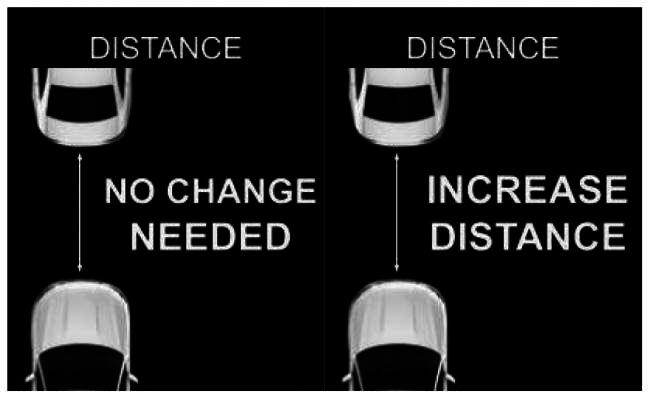

*Note*. A numerical factual value was presented instead of ‘xx’ for the Information condition.

Note that although the Information condition provided virtually the same speed information as the Control condition, given the presence of the speedometer, both conditions differ. Presenting the information in a dedicated feedback zone was expected to facilitate the information acquisition stage if the driver required support in that stage of the information-processing process. Moreover, explicitly monitoring behaviour can itself influence behaviour (McCambridge, Witton, and Elbourne 2014), making it important to observe the impact of this most basic level of feedback to understand the effects of more advanced feedback better.

For the Information condition, the feedback continuously provided the driver with their current speed (in km/h) and the distance to the car ahead (in metres), when applicable. For the conditions Assessment and Decision, the positive feedback was displayed at the start of the drive and would switch to the negative feedback if a threshold was exceeded. For speed, this threshold was when the speed was over the speed limit + 5.4 km/h (1.5 m/s), and for distance, the threshold was when the time headway (i.e. the distance headway divided by the ego vehicle’s speed) was less than 1.2 s. To prevent abrupt changes between positive and negative feedback, possibly leading to flashing images, a dead band was introduced by a second threshold. For the positive feedback to resume, the speed had to be below the speed limit + 0.5 m/s (1.8 km/h), and the time headway (THW) greater than 1.3 s.

#### 2.2.4. Recorded driving performance data

The two main variables of this experiment are speed and time headway (THW), presented as ‘distance’ to the participants. Both variables were recorded continuously throughout the drive. Based on that data, several dependent measures were computed, including average THW and speed, standard deviations of THW and speed, maximum and minimum THW and speed, and percentage of time below the recommended THW of 2 s or above the speed limit. To keep only relevant data, moments when the speed was < 3 km/h were removed, and the THW was analysed when it was below 100 s.

Furthermore, based on the speed and THW, feedback-related measures were computed: the percentage of time the positive feedback was displayed (i.e. when the behaviour was on the ‘correct’ side of the threshold) and the number of times negative feedback was triggered. These measures were also computed for the Control and Information conditions, although the presence of a threshold in those conditions was not made explicit to the participant.

#### 2.2.5. Self-reported data

Self-reports were used in addition to performance data. The demographic questions have already been detailed, together with their results, in Section 2.1, to describe the participant sample. Attitudes concerning the feedback options were also collected, and the mental effort and the situation awareness experienced during the drives were assessed. These variables were assessed immediately after each condition, but also at the end of the experiment after the participant had encountered all feedback options.

After each drive, opinions on the speed and THW feedback were collected with three indicators: whether participants found the feedback useful, whether they found it easy to understand, and whether they would like to have this kind of feedback in their car. After all the drives, two more items assessed attitudes: whether participants found the feedback useful was asked again, and whether they would intend to use the feedback if they could use any of the feedback options. For these five items, slider scales were used, ranging from 0 (not at all) to 100 (very much). At the end of the experiment, participants were also asked to rank the three types of feedback based on their preferences.

The subjective mental effort for each condition was assessed using the Rating Scale Mental Effort (RSME; Zijlstra and Van Doorn 1985) after each ride: participants could indicate their mental effort on a scale from 0 (absolutely no effort) to 150 (more than extreme effort). After all the rides, the subjective mental effort was assessed again per condition, using a slider scale ranging from 0 (no effort at all) to 100 (the biggest effort). Similarly, the situation awareness was assessed with three questions inspired by the Situational Awareness Rating Technique (SART; Taylor, Salas, and Dietz 2017) and adapted to fit the driving activity. For example, one question was, ‘How much were you concentrating on the road situations? Were you concentrating a lot (High) or a little (Low)?’, and was rated by participants from 1 (low) to 7 (high).

The questionnaire in its entirety is included in the Supplementary Material.

### 2.3. Procedure

After receiving information about the study, which specified that the study concerned feedback on driving, and after giving their informed consent, participants started the experiment with a practice drive, in which they had to complete the entire route presented in Figure 1. They were instructed to follow the signs to go to the city ‘Venekerk’, and were told that this route would take them through a rural area, a small village and then on a motorway and that they could stop the car at a parking area, after the exit lane of the motorway. They were also instructed to drive as they would drive in the real world. The feedback was not mentioned, nor were participants asked to follow the traffic rules. Upon completing the practice drive, and if they agreed to pursue the experiment, they completed a 15-minute questionnaire and proceeded with the experimental part of the study: four drives were completed on the same route again. Participants had to fill out a short questionnaire between each drive and after the four drives.

The order of the four conditions was randomised using a balanced Latin-square design (e.g. Edwards 1951). A Latin square design was originally generated, but to avoid carryover effects (Kim and Stein 2009), the conditions were also randomised by row and column, obtaining a balanced Latin-square design. Thirty different condition orders were then computed.

### 2.4. Data analysis

#### 2.4.1. Driving performance data

Two participants were excluded from the driving performance analysis: the first participant had missed a turn in one of the conditions, before making a U-turn to re-join the route, making the ride not comparable to the other three. The second participant showed aberrant and inconsistent performance across conditions, with for example a standard deviation of their average speed on the motorway of 25.1 km/h, against an average of 2.0 km/h for the other participants. This participant explained wanting to test the limits of the simulator by trying excessive speeds and accelerations and was therefore excluded. All the other participants were included in the analysis, even for example if their speed far exceeded the limits, as their behaviour remained consistent throughout the conditions. The final sample size for the driving performance data was *n* = 27.

The variables presented above (Section 2.2.4) were analysed using repeated measures analysis of variance (ANOVA) with feedback conditions as a factor. A p-value of .05 or under was considered significant, and effect sizes were interpreted according to Cohen’s guidelines (1988). When the repeated measures ANOVA yielded a significant result, further analyses were conducted using planned contrasts: indeed, when specific research questions and hypotheses are formulated a priori, contrasts can be used to answer these questions (Bewick, Cheek, and Ball 2004; Furr and Rosenthal 2003) and avoid Type III errors (i.e. ‘giving the right answer to the wrong question’, Haans 2018). The first question was whether one of the feedback conditions affected the variable compared to the baseline level, and was answered using simple contrast analysis (i.e. each feedback condition was compared to the control condition). The second question was whether, compared to the average behaviour, a condition yielded different results, and was answered using deviation contrasts (i.e. each condition was compared to the average of all four conditions).

These analyses were conducted for both speed and THW on the whole drive. Additionally, as depicted in Figure 1, specific sections of interest were identified. Speed was also analysed

1. in a rural area limited at 80 km/h (from 150 to 850 m after the start of the ride, titled ‘Section 1’),
2. in an urban area limited at 50 km/h (from 3600 to 4500 m, titled ‘Section 3’), and
3. on a motorway limited at 100 km/h (from 8500 to 10500 m, titled ‘Section 5’).

THW was also analysed

1. in an urban area (from 1500 to 2700 m, titled ‘Section 2’), and
2. on a motorway (from 6000 to 8000 m, titled ‘Section 4’), two sections where a car drove in front of the participant.

#### 2.4.2. Self-reported experiential data

All 29 participants were included in the analysis of the self-reports. Their opinions and attitudes were collected as described above, but the method of self-reporting led to 8.5% of missing data (167 missing values, out of a total of 1972). Missing data were imputed using the KNN imputation for the nearest neighbour row, a reliable method to estimate population parameters and allow data analysis techniques (Jadhav, Pramod, and Ramanathan 2019). Repeated measures ANOVAs were conducted on these results.

An acceptability score was computed for each feedback type based on the attitudes questions. This acceptability score is an average of the three scores to the questions asked immediately after each ride (usefulness, understandability, and whether participants would like to have this feedback) and the two scores to the questions asked at the end of the experiment (usefulness and intent to use). The acceptability score had a potential range of 0 to 100. To compare the acceptability of the different types of feedback, a two-way repeated measures ANOVA was used with feedback conditions (information, assessment, decision) and type of behaviour (speed, THW) as factors.

## 3. Results

### 3.1. Driving performance results

#### 3.1.1. Speed and THW on the whole drive

##### 3.1.1.1. Average speed and THW

**Figure 3.**
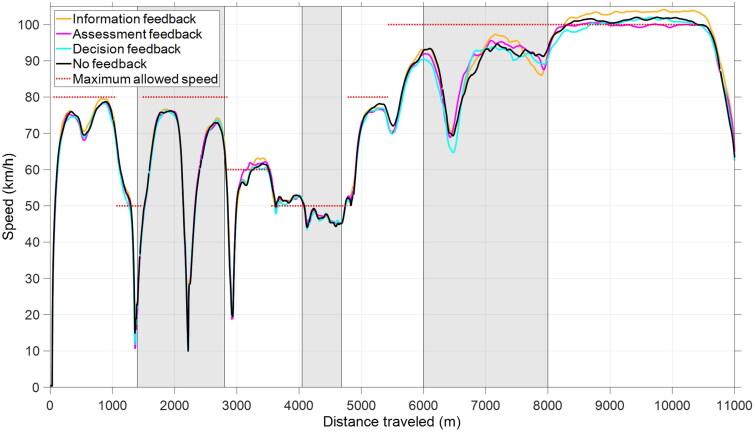
Mean speed across participants as a function of travelled distance, for each of the four experimental conditions. *Note.* The horizontal dotted lines represent the speed limits on that portion of the route. The grey-shaded backgrounds represent portions of the route where car-following took place.

On average, participants drove 67.5 km/h (*SD* = 4.6), and more specifically, on average 67.7 km/h (*SD* = 4.4) in the Control condition, 67.9 km/h (*SD* = 4.6) in the Information condition, 67.7 km/h (*SD* = 4.9) in the Assessment condition and 66.8 km/h (*SD* = 4.3) in the Decision condition. The repeated measure ANOVA revealed no significant difference (*F*(3,78) = 1.60, *p* = .196, η^2^ = .058) between conditions, yet indicate a small to medium effect size for a repeated-measure ANOVA. The speed in all conditions is plotted against the travelled distance driven in Figure 3.

The average THW was not significantly different across conditions (*F*(3,78) = 1.68, *p* = .178, η^2^ = .061): 4.6 s (*SD* = 1.0) in the Control condition, 4.7 s (*SD* = 1.5) in the Information condition, 4.8 s (*SD* = 1.2) in the Assessment condition and 5.0 s (*SD* = 1.2) in the Decision condition.

##### 3.1.1.2. Standard deviation of speed and THW

The participants’ average *SD* of speed was significantly different across conditions (*F*(3,78) = 4.19, *p* = .008, η^2^ = .139). The average *SD* of speed was 24.6 km/h (*SD* = 1.5) in the Control condition, 25.0 km/h (*SD* = 1.1) in the Information condition, 24.8 km/h (*SD* = 1.3) in the Assessment condition and 24.2 km/h (*SD* = 1.1) in the Decision condition. The simple contrast analysis revealed no significant differences, while the deviation contrasts analysis revealed that the *SD* of speed was significantly higher (0.37 km/h) in the Information condition than in the overall average *SD* (*t*(78) = 2.57, *p* = .012) and that the *SD* of speed was lower (0.43 km/h) in the Decision condition (*t*(78) = −3.03, *p* = .003).

The standard deviations of THW were not statistically significantly different across conditions (*F*(3,78) = 0.06, *p* = .980, η^2^ = .002), with an average *SD* of 5.1 s in the Control, Information and Assessment conditions and 5.2 s in the Decision condition.

##### 3.1.1.3. Maximum speed and minimum THW

Differences were found in the maximum speed (*F*(3,78) = 3.87, *p* = .012, η^2^ = .130). The simple contrast analysis revealed no significant difference, and the deviation contrast analysis revealed that the maximum speed was higher in the Information condition (*t*(78) = 2.91, *p* = .005) with an average maximum speed of 106.9 km/h (*SD* = 7.2), and lower in the Decision condition (*t*(78) = −2.63, *p* = .010) with an average maximum speed of 104.1 km/h (*SD* = 5.0).

No significant differences were found in minimum THW (*F*(3,78) = 0.14, *p* = .936, η^2^ = .005) across conditions.

##### 3.1.1.4. Comparison to the limits

The comparison between the percentage of time above the speed limit differed significantly, *F*(3,78) = 3.96, *p* = .011, η^2^ = .132. Participants were above the speed limit for 22.1% (*SD* = 15.3) of the drive in the Control condition, 26.1% (*SD* = 16.0) in the Information condition, 24.3% (*SD* = 16.0) in the Assessment condition and 20.1% (*SD* = 13.0) in the Decision condition. The simple contrast analysis indicated one significant difference: the time spent above the speed limit was significantly higher (4.1%) in the Information condition than in the Control condition (*t*(78) = 2.18, *p* = .033). The deviation contrast analysis revealed that the percentage of time above the speed limit was higher in the Information condition (*t*(78) = 2.60, *p* = .011), and lower in the Decision condition (*t*(78) = −2.67, *p* = .009).

**Figure 4.**
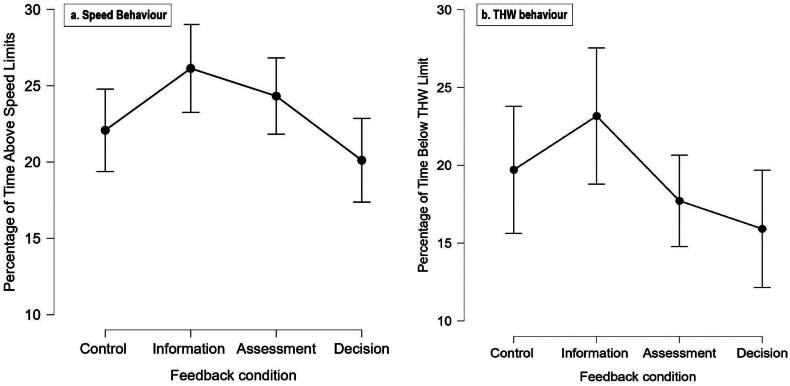
Percentage of time exceeding the limits depending on the feedback condition and the behaviour type.

The percentage of time below the recommended THW (i.e. under 2 s) yielded a significant difference in the repeated measures ANOVA (*F*(3,78) = 2.78, *p* = .047, η^2^ = .097); participants were below the THW limit for 19.7% (*SD* = 16.0) of the drive in the Control condition, 23.2% (*SD* = 19.6) in the Information condition, 17.7% (*SD* = 15.7) in the Assessment condition and 15.9% (*SD* = 11.5) in the Decision condition. The simple contrast analysis revealed no statistically significant differences, and the deviation contrast analysis revealed that the percentage of time below the limit was significantly higher (4.0%) in the Information condition (*t*(78) = 2.50, *p* = .014) than the average of all conditions. These results are illustrated in Figure 4, with normalised 95% confidence intervals error bars (Morey 2008).

#### 3.1.2. Speed and THW in specific sections

Speed was investigated in three sections: a rural area (‘Section 1’), which yielded no significant differences (*F*(3,78) = 1.14, *p* = .337, η^2^ = .042), an urban area (‘Section 3’), which also yielded no significant differences (*F*(3,78) = 1.52, *p* = .215, η^2^ = .055), and a motorway area (‘Section 5’), which led to significant differences in the average speed (*F*(3,78) = 5.15, *p* = .003, η^2^ = .165). On this particular section of the motorway, the average speed was 100.1 km/h (*SD* = 4.9) in the Control condition, 101.5 km/h (*SD* = 3.8) in the Information condition, 99.9 km/h (*SD* = 4.1) in the Assessment condition, and 99.0 km/h (*SD* = 2.9) in the Decision condition. The simple contrast analysis indicated one significant difference: the average speed was significantly higher (1.3 km/h) in the Information condition than in the Control condition (*t*(78) = 2.04, *p* = .044). Deviation contrast analysis indicated that the average speed was significantly higher (1.4 km/h) in the Information condition than the average (*t*(78) = 3.42, *p* = .001) and 1.2 km/h lower in the Decision condition (*t*(78) = −2.93, *p* = .005).

The analysis of the average THW revealed no significant differences, neither in the rural area (‘Section 2’; *F*(3,78) = 1.43, *p* = .239, η^2^ = .052) nor in the motorway area (‘Section 4’; *F*(3,78) = 0.49, *p* = .687, η^2^ = .019). The average THW was 6.4 s for Sections 2 and 3.7 for Section 4.

#### 3.1.3. Other variables

##### 3.1.3.1. Percentage of negative feedback

The comparison of the percentages of time participants received negative feedback on speed revealed no significant differences (*F*(3,78) = 2.57, *p* = .060, η^2^ = .090). Note that although participants in the Control condition received no feedback and those in the Information condition received only neutral feedback, we estimated the virtual negative feedback that hypothetically would have been provided in these conditions using the same thresholds as used for the negative feedback in the Assessment and Decision conditions. Participants received, on average, negative feedback for 8.2% (*SD* = 11.0) of the drive in the Control condition, 9.0% (*SD* = 11.1) in the Information condition, 6.3% (*SD* = 9.1) in the Assessment condition and 4.9% (*SD* = 4.7) in the Decision condition.

The percentage of negative feedback for the THW was also found to be not statistically different across conditions (*F*(3,78) = 2.01, *p* = .119, η^2^ = .072). Participants received negative feedback for 6.8% (*SD* = 12.7) of the drive in the Control condition, 7.3% (*SD* = 13.4) in the Information condition, 4.6% (*SD* = 9.7) in the Assessment condition and 3.5% (*SD* = 3.3) in the Decision condition.

##### 3.1.3.2. Number of triggers of negative feedback

The number of times the speed feedback turned negative was not significantly different between conditions (*F*(3,78) = 0.33, *p* = .802, η^2^ = .013). On average, the feedback turned negative 4.0 times (*SD* = 3.0) in the Control condition, 4.4 times (*SD* = 3.3) in the Information condition, 4.3 times (*SD* = 3.1) in the Assessment condition and 4.1 times (*SD* = 2.8) in the Decision condition.

Similarly, the number of times the THW feedback turned negative was not significantly different across conditions (*F*(3,78) = 0.50, *p* = .685, η^2^ = .019). On average, the feedback turned negative 2.1 times (*SD* = 1.6) in the Control condition, 2.2 times (*SD* = 1.3) in the Information condition, 2.0 times (*SD* = 1.6) in the Assessment condition and 2.3 times (*SD* = 1.3) in the Decision condition.

### 3.2. Self-reported results

#### 3.2.1. Attitudes and acceptability of the feedback

Attitudes about the feedback were collected both immediately after participants experienced the feedback condition (i.e. after each drive) and after the experiment (i.e. after all the conditions were completed). These two different measurement moments are differentiated in the results as ‘Immediate attitudes’ (i.e. after experiencing each condition) and ‘Post-experiment attitudes’ (i.e. after experiencing all conditions). The mean scores and standard deviations of these items are presented in Table 3 for the feedback on Speed and in Table 4 for the feedback on THW.

**Table 3.** Mean scores and standard deviations of attitudes items (range 0–100) on the speed feedback.

Feedback condition	Information	Assessment	Decision
*Immediate attitudes (on Speed Feedback)*			
The information was useful	58.3 (31.7)	65.3 (21.3)	50.1 (27.3)
The information was easy to understand*	92.8* (9.7)	88.0 (18.5)	81.2* (23.3)
I would like to have this kind of information available in my own car**	64.3^a^* ^b^** (34.0)	44.4^a^* (30.2)	39.9^b^** (28.0)
*Post-experiment attitudes (on Speed Feedback)*			
The information was useful*	67.4* (31.9)	58.8 (25.4)	49.5* (26.2)
Assuming that all feedback options were available in your car, intent to use feedback***	69.2^a^**,^b^*** (31.4)	45.6^a^** (29.8)	39.9^b^*** (27.4)
*Acceptability score (on Speed Feedback)*			
Average of the five prior items**	70.4** (22.0)	60.4 (20.3)	52.1** (17.7)

*Note*. Statistical differences were investigated with RM ANOVA and are indicated with.

* When *p* < .05, ** when *p* < .01, and *** when *p* < .001.

**Table 4.** Mean scores and standard deviations of attitudes items (range 0–100) on the THW feedback.

Feedback condition	Information	Assessment	Decision
*Immediate attitudes (on THW Feedback)*			
The information was useful	41.8 (28.5)	43.6 (21.6)	50.2 (32.6)
The information was easy to understand	82.1 (28.1)	92.1 (10.9)	80.1 (25.0)
I would like to have this kind of information available in my own car	40.2 (29.5)	40.4 (29.8)	50.5 (32.7)
*Post-experiment attitudes (on THW Feedback)*			
The information was useful	45.6 (33.7)	47.9 (27.5)	52.7 (30.2)
Assuming that all feedback options were available in your car, intent to use feedback	38.4 (34.2)	41.6 (28.0)	42.8 (33.3)
*Acceptability score (on THW Feedback)*			
Average of the five prior items	49.6 (25.3)	53.1 (17.5)	55.3 (21.5)

*Note*. No significant differences were found between conditions.

In terms of speed-related feedback, the Information feedback was preferred overall, with an average acceptability score of 70.4, against 60.4 and 52.1 for the Assessment and Decision feedback. The Information feedback received the highest rating on understandability, desire to have the feedback, intent to use if all options were available and usefulness when asked at the end of the experiment.

Regarding distance-related feedback, no such pattern was found as no condition stood out as preferred. Acceptability scores averaged around 50, in the middle of the scale from 0 to 100; indicating in this context that participants are neither positive nor negative about the THW feedback. The Decision feedback was rated slightly higher on its usefulness and desire to have the feedback.

Acceptability scores were further analysed with a two-way repeated measures ANOVA, which indicated a significant effect for one of the factors: the type of behaviour targeted yielded differences (*F*(1,28) = 12.9, *p* = .001, η^2^ = .049), but not the condition (*F*(2,56) = 0.89, *p* = .418, η^2^ = .019). Feedback, regardless of the condition, was preferred when it related to speed than THW (*p* = .001, 95% C.I. = [3.6; 13.1]).

**Figure 5.**
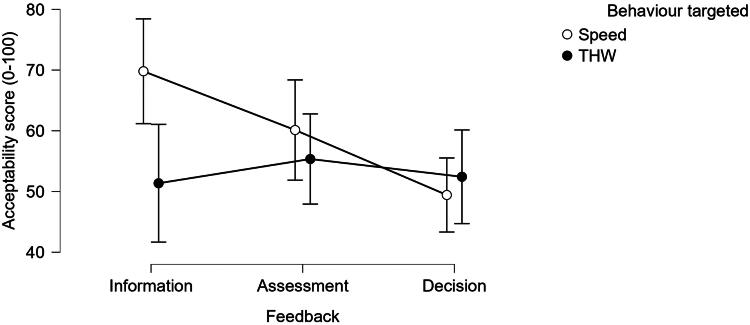
Acceptability scores (range 0–100) depending on the condition and the type of behaviour.

The interaction effect was also significant: *F*(2,56) = 12.1, *p* < .001, η^2^ = .068. The post hoc analysis of the interaction effect, using the Bonferroni correction, revealed that three pairs are significantly different: (1) the Information feedback was preferred when it related to speed than to THW (*p* < .001, 95% C.I. = [9.8; 31.8]), (2) the Information feedback on speed was preferred to the Assessment feedback on THW (*p* = .032, 95% C.I. = [0.8; 33.8]) and (3) when it related to speed, the Information feedback was preferred to the Decision feedback (*p* = .014, 95% C.I. = [2.1; 34.4]). The acceptability scores are illustrated in Figure 5, with normalised 95% confidence intervals error bars (Morey 2008).

The average rank, from 1 (most preferred) to 3 (least preferred), was 1.57 (*SD* = 0.68) for the Speed feedback and 2.48 (*SD* = 1.33) for the THW feedback in the Information condition. In the Assessment condition, it was 2.24 (*SD* = 0.94) for the Speed feedback and 2.38 (*SD* = 1.36) for THW feedback, and in the Decision condition, the ranks were 2.19 (*SD* = 0.68) for Speed feedback and 2.67 (*SD* = 0.73) for THW feedback.

#### 3.2.2. Impact of feedback on mental effort and situation awareness

The mental effort, evaluated on the RSME ranging from 0 to 150 directly after each ride, resulted in scores of 45.7 (*SD* = 25.0) in the Control condition, 45.9 (*SD* = 23.9) in the Information condition, 45.5 (*SD* = 23.6) in the Assessment condition and 46.7 (*SD* = 23.5) in the Decision condition. These results are not statistically different (*F*(3,84) = 0.08, *p* = .971, η^2^ = .003). At the end of the experiment, the mental effort was assessed again, on a scale from 0 to 100: 43.2 (*SD* = 22.8) in the Control condition, 45.2 (*SD* = 23.1) in the Information condition, 40.7 (*SD* = 24.2) in the Assessment condition and 41.0 (*SD* = 26.2) in the Decision condition, all found to not be statistically different (*F*(3,84) = 0.84, *p* = .476, η^2^ = .029).

The situation awareness was first assessed directly after each ride, on a 7-point Likert scale from 1 (least aware) to 7 (most aware). The results are not statistically different (*F*(3,84) = 0.34, *p* = .798, η^2^ = .012): the average score was 4.7 (*SD* = 0.9) in the Control condition, 4.8 (*SD* = 0.8) in the Information condition, 4.7 (*SD* = 0.7) in the Assessment condition, and 4.7 (*SD* = 0.8) in the Decision condition. Similar to the mental effort variable, the situation awareness was assessed again at the end of the experiment with a scale from 0 to 100: 64.2 (*SD* = 24.7) in the Control condition, 58.4 (*SD* = 24.2) in the Information condition, 54.1 (*SD* = 23.4) in the Assessment condition and 54.5 (*SD* = 24.7) in the Decision condition; which also yielded no statistical difference (*F*(3,81) = 2.71, *p* = .051, η^2^ = .091).

## 4. Discussion

This driving simulator study aimed to investigate the stage of information processing required when receiving feedback on driver behaviour. Specifically, we sought to determine whether providing a piece of information alone (Stage 1 feedback), an assessment of that information (Stage 2 feedback), or a decision based on that assessment (Stage 3 feedback) would yield different driving outcomes, and which approach would better support drivers in their tasks. This research interest was grounded in the assumption that providing feedback affects behaviour, as was observed for example by Abrahamse et al. (2005) on household energy conservation and De Waard, Van der Hulst, and Brookhuis (1999) on traffic violations.

The first result of the study is the similarity of behaviours in all four conditions, i.e. the control as well as three feedback conditions. Providing feedback had no apparent effect on the two behaviours of interest, speed and time headway (THW). Still, other behavioural variables yielded differences between feedback conditions. This was the case, for example, for the standard deviation of speed, the maximum speed, the time above speed limits, time under the recommended THW, and the speed on the motorway section. For most of these measures, it turned out that the Information condition yielded worse behaviour and the Decision condition yielded better behaviour. These two main findings – the non-significant effect of feedback conditions on speed and THW and the significant effect on other behavioural variables – will be discussed here, along with results from the self-reports.

The first result of the study is the apparent null effect of feedback on speed and THW. This finding points to the difficulty of inducing observable behaviour change with feedback. Even though both target behaviours did not differ significantly across conditions, the eta squared values (η^2^ = .058 for speed and η^2^ = .061 for THW) indicate medium effects (Cohen 1988), and suggest that a larger sample size may be needed to confirm the results. Additionally, a significant change in speed was observed on the motorway with a large effect size (η^2^ = .165) and a maximum difference in mean speed of 2.5 km/h between conditions. Thus, providing feedback had a limited but non-negligible effect: a change of 2.5 km/h, especially in a zone limited to 100 km/h, has an impact on crash reduction (approximately 7.5% reduction according to Mazureck and Van Hattem 2006). These relatively limited effects may be unexpected given the results of a recent questionnaire study by Ge, Luo, and Qu (2023), in which a significant difference in self-reported behavioural intentions was observed among 110 participants between feedback conditions similar to those used in this study. Thus, it seems that neither behavioural intentions nor feedback alone may consistently lead to meaningful behavioural change.

The limited effect of feedback alone, i.e. without specific instructions or incentives, has been noted before. For example, Marciano, Setter, and Norman (2015) investigated the effect of overt and covert speed cameras and of immediate feedback and observed no improvement in speed due to immediate feedback alone. Furthermore, Mullen, Maxwell, and Bédard (2015) conducted an on-road study in which they examined the effect of feedback and of a token economy (i.e. monetary incentive) on speeding behaviour. The results not only showed no difference between the feedback-only and control conditions but also indicated similar and better behaviours in the token economy coupled with feedback and token-only conditions. In other words, providing an incentive, even without feedback, yielded behaviour change, whereas providing feedback without an incentive did not. The same conclusion was reached by Bolderdijk et al. (2011) in a study investigating the effect of a Pay-As-You-Drive (PAYD) scheme on speed choice. They found that a combination of feedback and incentives was effective in reducing speeds. However, they reported that the effect was most likely due to the incentive alone, as the majority of their participants did not log in to the website to access their feedback. This suggests that participants received virtually no feedback, and the incentive alone was sufficient to change their behaviour. Considering that many studies on the effect of (real-time) feedback on driving performance also include incentives or rewards (e.g. Dijksterhuis et al. 2015, 2016; Masello et al. 2023; Mazureck and Van Hattem 2006), it seems reasonable to infer that the effect of incentives on behaviour could have been misattributed to the potential effect of feedback. Whether the lack of studies involving real-time feedback alone is due to publication bias or limited research interest remains to be determined.

The modest effect of feedback, combined with the more pronounced effect of incentives, provides grounds for considering how drivers can be guided towards behaviour change. Based on the studies from Mullen, Maxwell, and Bédard (2015) and Bolderdijk et al. (2011), as well as the primary result of the current simulator experiment, it seems that providing external motivation leads to behavioural change. In contrast, providing feedback that merely assists drivers in self-assessment seems to only have a limited effect. This statement is further supported by the transient nature of most safety interventions: behaviours tend to improve while incentives are present but regress once the external motivation is removed, indicating a lack of long-term learning (see e.g. Mazureck and Van Hattem 2006). Speeding should therefore be considered more of a motivational issue than a capability one, and the same logic could be extended to other risky driving behaviours. As an example, Pampel et al. (2018) brought to light that people know how to drive eco-friendly and can do so when prompted, but they would return to ‘everyday’ driving after some time or when their mental workload increases. It could be argued that a similar pattern exists for safe driving: people know how to drive safely, but without ongoing incentives or external motivation, this behaviour is not sustained.

The limited behavioural change observed from feedback alone is understandable from this perspective. It can be hypothesised that speed and THW are aspects of driving that are not challenging for drivers to monitor and assess; Those behaviours are not controlled consciously all the time (see e.g. Kahneman 2011) but are performed in everyday/habitual ‘auto-pilot’ mode. This hypothesis is confirmed by the fact that participants indicated not having difficulties assessing their speed and THW behaviour. Thus, it can be argued that drivers follow a long-established mental model for speed and THW, which is only minimally influenced by informative feedback alone. And, similar to what was observed by Pampel et al. (2018), when specifically requested or incentivised, drivers *can* control speed and THW behaviour to fit their specific goals better.

When feedback conditions yielded different behaviour, it most often followed a certain pattern: the Information condition yielded worse behaviour (specifically on the standard deviation of speed, maximum speed, time spent above the speed limits and under the THW recommendation), the Assessment condition did not differ even once from the Control condition, and the Decision condition yielded better behaviour (specifically on the standard deviation of speed, maximum speed and the time spent above the speed limits). Better or worse behaviours are determined as follows: a higher speed SD is considered more dangerous as greater fluctuations in speed reduce safety margins, and a higher maximum speed is also considered more dangerous, as is more time spent above speed or THW limits. The differences found between feedback conditions, along with the pattern observed, suggest an effect of the stages of information processing.

The nuances of this pattern of results are as follows. First, with respect to the results of the Assessment condition, providing drivers with an assessment of their behaviour did not have any effect compared to a control condition. While speed limits could be inferred from the speed signs in the environment, the Assessment condition continuously provided drivers with a judgement of whether they were speeding or not. The null result seems to point to the fact that their own assessment (i.e. comparing their speed to the speed limits) was already effective. In fact, this result is in line with elements developed earlier in this discussion: it indicates that the participants already knew whether their behaviour was good or bad, perhaps in the same way that people know how to drive in an eco-friendly manner (Pampel et al. 2018). Second, the Information condition yielded worse behaviour, which could be an example of behavioural adaptation (Smiley and Rudin-Brown 2020): it can be hypothesised that providing a numerical value for the speed changed the basis on which participants made their assessment, possibly making them feel safer, and causing an increase in dangerous behaviour. Third and last, the Decision condition was the most effective in inducing positive behaviour change and could be explained by two mechanisms. It could indicate a failure in linking dangerous behaviour to a need for behaviour change: informing participants that their behaviour was non-compliant did not have any effect compared to not informing them about this (as was done in the Assessment condition), but *instructing* them to adopt a specific behaviour when their behaviour was non-compliant had some effect. It could also be evidence of more social phenomena, such as social desirability or compliance with instructions, as the feedback made clear what was expected from the participants in the Decision condition.

In addition to differences in stages of information processing, the feedback conditions differed in terms of modality (i.e. numerical values, icons, text), which inherently can affect behaviour, by requiring more or less interpretation time, causing distraction, or even confusion. While this may be true, the feedback options were designed to be simple and easy to understand (as assessed through a pre-study questionnaire) and were rated as easy to understand by the participants. The speed Decision feedback was the least easy to understand but reduced speed variability; Considering higher speed variability is an indicator of distraction (Papantoniou, Papadimitriou, and Yannis 2017), this result suggests that the speed Decision feedback was not distracting. Future research could measure gaze behaviour to investigate attention allocation and better understand modality differences.

Interestingly, the condition that produced the best behavioural results also yielded the worst attitudinal results: while all feedback conditions received similar ratings for the THW variable, for the speed variable, the results differed with the Information condition being the preferred feedback and the Decision condition the least liked. This suggests a trade-off between experiential and instrumental outcomes, similar to the results of Degirmenci and Breitner (2023). In informal discussions following the experiment, participants primarily expressed their dislike of being told what to do, with some mentioning that they felt more capable than their car at deciding the best behaviour to adopt. This element could be a shortcoming of Decision-oriented feedback: the credibility of the feedback and its issuer are main predictors of effectiveness (Poulos and Mahony 2008). Another potential shortcoming could be the exclusion of the driver from the decision-making process, which could possibly reduce situation awareness (De Winter et al. 2014), although the feedback in this experiment did not statistically significantly impact the participants’ self-reported situation awareness. In fact, the feedback seems to have neither negative nor positive consequences in terms of mental effort and situation awareness.

It should be noted that this experiment was conducted on a simulator which implies certain limitations: results obtained in simulated environments may not always be predictive of real-world behaviour (Wynne, Beanland, and Salmon 2019). However, speed and speed variation are often found to be equivalent (absolute validity) or to show similar patterns (relative validity) between a simulation and a real drive (Godley, Triggs, and Fildes 2002; Wynne, Beanland, and Salmon 2019); and relative validity was observed for headway behaviour on five different simulators (Klüver et al. 2016). The validity of the present results could be ensured by replicating the study in real-world settings; yet, the primary finding of this study (i.e. the relatively small effect of feedback) seems consistent with literature about on-road driving, as discussed previously.

## 5. Conclusion

This experiment entails two main findings: (1) the limited effect that feedback alone has on speed and THW, pointing to risky behaviour being a motivational issue rather than a capability one, and (2) the importance of the stage of information processing of the feedback. From an ergonomics viewpoint, it seemed that suggesting a behaviour was the best approach (Forzy 2004) as a way to keep the driver in the control loop. However, the main drawback of this approach is that it does not directly affect motivation. Future research should focus on identifying ways to encourage safer behaviour, either through changing risk perception (possibly via feedback) or through incentives, as incentives have been proven more effective. Identifying risks and risky behaviour does not appear to be an issue for drivers; however, risks are too often considered acceptable on the roads (see e.g. Molin and Brookhuis (2007), who discussed the influence of beliefs regarding speeding on the acceptability of Intelligent Speed Adaptation).

The recommendations for future safety interventions are: (1) to assist drivers in associating road risks with the need for behavioural change, either by altering their risk perceptions and beliefs or by providing external motivation for this behavioural change; and (2) to suggest a specific behaviour to adopt when providing feedback, as this has been shown to be the most effective stage of information processing for influencing drivers.

## Supplementary Material

Supplemental Material
