# Supplementary material for: Information, assessment, or decision: a driving simulator study on the effect of real-time feedback based on information-processing stages
Source: Ergonomics. 2025 Jul 14:1–16. Online ahead of print. doi: 10.1080/00140139.2025.2477624 (PMC12585109; doi:10.1080/00140139.2025.2477624)
Supplement: Supplemental Material [file TERG_A_2477624_SM8575.docx]

Simulator study (feedback)

Start of Block: START OF THE SURVEY(to be completed by experimenter!)

participant_ID (filled by experimenter)

Participant number

________________________________________________________________

order_conditions (filled by experimenter)

Order of conditions

________________________________________________________________

End of Block: START OF THE SURVEY(to be completed by experimenter!)

Start of Block: info

info The following questions should be completed by the participant

End of Block: info

Start of Block: REQUIREMENTS

consent_form Thank you for agreeing to participate in this study. Your participation in this research is entirely voluntary. You have the right to withdraw at any point during the study, for any reason, and without facing any negative consequences. The study should take approximately 1h30. 

If you have any questions at any time, you can ask the experimenter present with you, or send an email to a.picco@rug.nl. 

By clicking the consent button below, you acknowledge that your participation in the study is voluntary, that you are at least 18 years of age, and that you are aware that you may choose to terminate your participation in the study at any time and for any reason.

- Yes, I consent
- No, I do not consent

Skip To: End of Survey If Thank you for agreeing to participate in this study. Your participation in this research is entir... = No, I do not consent

instructions After answering the questions on the page displayed, you can click on the arrow (à) in the bottom right corner, to go to the next page. Your progress will be displayed via the red bar at the top of the screen. 

This questionnaire contains multiple parts, please follow the instructions that will appear on the screen.

driving_licence Are you currently in possession of a driving licence?

- Yes
- No

Skip To: End of Survey If Are you currently in possession of a driving licence? = No

End of Block: REQUIREMENTS

Start of Block: DEMOGRAPHICS (before rides)

| 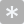 |
| --- |

age How old are you?

________________________________________________________________

nationality What is your nationality?

________________________________________________________________

gender How do you describe yourself?

- Male
- Female
- Non-binary / third gender
- Prefer to self-describe __________________________________________________
- Prefer not to say

| 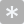 |
| --- |

age_driving_licence At what age did you get your first driving licence?

________________________________________________________________

professional_driver Are you a professional driver?

- Yes
- No

primary_transport What is your primary mode of transportation, including during your working time?

- Walking
- Cycling
- Public transportation
- Private vehicle (car)
- Private vehicle (other: e.g., motorcycle, motor scooter)
- Other __________________________________________________

| 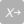 |
| --- |

kilometres_in_year About how many kilometres do you usually drive your car in a year, including during your working time?

- 0 km
- 1 to 1000 km
- 1001 to 5000 km
- 5001 to 10 000 km
- 10 001 to 20 000 km
- 20 001 to 30 000 km
- 30 001 to 50 000 km
- 50 001 to 100 000 km
- More than 100 000 km

End of Block: DEMOGRAPHICS (before rides)

Start of Block: SELF-ASSESSMENT

enjoyment_driving What is your opinion regarding the statement "I enjoy driving a car"?

- Strongly disagree
- Disagree
- Somewhat disagree
- Neither agree nor disagree
- Somewhat agree
- Agree
- Strongly agree

quality_driving What is your opinion regarding the statement "I am a good driver"?

- Strongly disagree
- Disagree
- Somewhat disagree
- Neither agree nor disagree
- Somewhat agree
- Agree
- Strongly agree

better_than_average What is your opinion regarding the statement "I am better than the average driver"?

- Strongly disagree
- Disagree
- Somewhat disagree
- Neither agree nor disagree
- Somewhat agree
- Agree
- Strongly agree

improvement_driving What is your opinion regarding the statement "When it comes to my driving ability, there is still room or improvement"?

- Strongly disagree
- Disagree
- Somewhat disagree
- Neither agree nor disagree
- Somewhat agree
- Agree
- Strongly agree

acceptance_speeding What is your opinion regarding the statement "If you are a good driver it is acceptable to drive a little faster"?

- Strongly disagree
- Disagree
- Somewhat disagree
- Neither agree nor disagree
- Somewhat agree
- Agree
- Strongly agree

acceptance_yellow What is your opinion regarding the statement "It is acceptable to drive when traffic lights change from green to yellow"?

- Strongly disagree
- Disagree
- Somewhat disagree
- Neither agree nor disagree
- Somewhat agree
- Agree
- Strongly agree

End of Block: SELF-ASSESSMENT

Start of Block: TECHNOLOGY AFFINITY (before rides)

tech_affinity1 What is your opinion regarding the statement "I like testing the functions of new technical systems"?

- Strongly disagree
- Disagree
- Somewhat disagree
- Neither agree nor disagree
- Somewhat agree
- Agree
- Strongly agree

tech_affinity2 What is your opinion regarding the statement "I predominantly deal with technical systems because I have to"?

- Strongly disagree
- Disagree
- Somewhat disagree
- Neither agree nor disagree
- Somewhat agree
- Agree
- Strongly agree

tech_affinity3 What is your opinion regarding the statement "I try to make full use of the capabilities of a technical system"?

- Strongly disagree
- Disagree
- Somewhat disagree
- Neither agree nor disagree
- Somewhat agree
- Agree
- Strongly agree

tech_affinity4 What is your opinion regarding the statement "I enjoy spending time becoming acquainted with a new technical system"?

- Strongly disagree
- Disagree
- Somewhat disagree
- Neither agree nor disagree
- Somewhat agree
- Agree
- Strongly agree

tech_affinity5 What is your opinion regarding the statement "I like using Advanced Driver-Assistance Systems (e.g., adaptive cruise control, blind spot monitor, parking sensor)"?

- Strongly disagree
- Disagree
- Somewhat disagree
- Neither agree nor disagree
- Somewhat agree
- Agree
- Strongly agree

tech_affinity6 What is your opinion regarding the statement "I trust the technology of Advanced Driver-Assistance Systems (e.g., adaptive cruise control, blind spot monitor, parking sensor)"?

- Strongly disagree
- Disagree
- Somewhat disagree
- Neither agree nor disagree
- Somewhat agree
- Agree
- Strongly agree

End of Block: TECHNOLOGY AFFINITY (before rides)

Start of Block: info

info2 This is the end of the first section of the questionnaire. 

You can give back the laptop to the experimenter and we will start with the driving part. 

End of Block: info

Start of Block: MW and SA / after ride 1

condition_t1 Condition (filled by experimenter)

________________________________________________________________

condition_t1_control (filled by experimenter) was this the control condition?

- yes
- no

| Page Break |  |
| --- | --- |

instruction_t1 Based on the scale presented below, how would you rate your mental effort during this ride?

MW_pic_t1

| 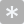 |
| --- |

MW_t1 Response between 0 and 150

________________________________________________________________

| Page Break |  |
| --- | --- |

SA_t1_q1 How much were you concentrating on the road situations? Were you concentrating a lot (High) or a little (Low)?

|  | 1 | 2 | 3 | 4 | 5 | 6 | 7 |
| --- | --- | --- | --- | --- | --- | --- | --- |

| 1 is Low, 7 is High |  |
| --- | --- |

SA_t1_q2 How much was your attention divided during the drive? Were you concentrating on many aspects of the road situations (High) or focused on only one (Low)?

|  | 1 | 2 | 3 | 4 | 5 | 6 | 7 |
| --- | --- | --- | --- | --- | --- | --- | --- |

| 1 is Low, 7 is High |  |
| --- | --- |

SA_t1_q3 How much mental capacity did you have to spare during the drive? Did you have enough capacity to be able to attend to another task (High) or not (Low)?

|  | 1 | 2 | 3 | 4 | 5 | 6 | 7 |
| --- | --- | --- | --- | --- | --- | --- | --- |

| 1 is Low, 7 is High |  |
| --- | --- |

| Page Break |  |
| --- | --- |

Display This Question:

If (filled by experimenter) was this the control condition? = no

At_t1_speed You were presented with information on your **speed** during this ride. What is your opinion regarding the following statements, from 0 "not at all" to 100 "very much"?

|  | 0 | 10 | 20 | 30 | 40 | 50 | 60 | 70 | 80 | 90 | 100 |
| --- | --- | --- | --- | --- | --- | --- | --- | --- | --- | --- | --- |

| "The information on speed was useful" | 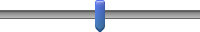 |
| --- | --- |
| "The information on speed was easy to understand" | 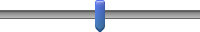 |
| "I would like to have this kind of information available in my own car" | 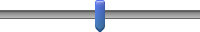 |

Display This Question:

If (filled by experimenter) was this the control condition? = no

At_t1_distance You were presented with information on the **distance to the car ahead**during this ride. What is your opinion regarding the following statements, from 0 "not at all" to 100 "very much"?

|  | 0 | 10 | 20 | 30 | 40 | 50 | 60 | 70 | 80 | 90 | 100 |
| --- | --- | --- | --- | --- | --- | --- | --- | --- | --- | --- | --- |

| "The information on distance was useful" | 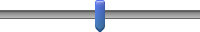 |
| --- | --- |
| "The information on distance was easy to understand" | 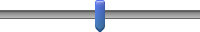 |
| "I would like to have this kind of information available in my own car" | 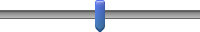 |

End of Block: MW and SA / after ride 1

Start of Block: info 1

info3 This is the end of this section of the questionnaire. 

You can give back the laptop to the experimenter and we will resume with the driving part. 

End of Block: info 1

Start of Block: MW and SA / after ride 2

condition_t2 Condition (filled by experimenter)

________________________________________________________________

condition_t2_control (filled by experimenter) was this the control condition?

- yes
- no

| Page Break |  |
| --- | --- |

instruction_t2 Based on the scale presented below, how would you rate your mental effort during this ride?

MW_pic_t2

| 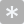 |
| --- |

MW_t2 Response between 0 and 150

________________________________________________________________

| Page Break |  |
| --- | --- |

SA_t2_q1 How much were you concentrating on the road situations? Were you concentrating a lot (High) or a little (Low)?

|  | 1 | 2 | 3 | 4 | 5 | 6 | 7 |
| --- | --- | --- | --- | --- | --- | --- | --- |

| 1 is Low, 7 is High |  |
| --- | --- |

SA_t2_q2 How much was your attention divided during the drive? Were you concentrating on many aspects of the road situations (High) or focused on only one (Low)?

|  | 1 | 2 | 3 | 4 | 5 | 6 | 7 |
| --- | --- | --- | --- | --- | --- | --- | --- |

| 1 is Low, 7 is High |  |
| --- | --- |

SA_t2_q3 How much mental capacity did you have to spare during the drive? Did you have enough capacity to be able to attend to another task (High) or not (Low)?

|  | 1 | 2 | 3 | 4 | 5 | 6 | 7 |
| --- | --- | --- | --- | --- | --- | --- | --- |

| 1 is Low, 7 is High |  |
| --- | --- |

| Page Break |  |
| --- | --- |

Display This Question:

If (filled by experimenter) was this the control condition? = no

At_t2_speed You were presented with information on your **speed** during this ride. What is your opinion regarding the following statements, from 0 "not at all" to 100 "very much"?

|  | 0 | 10 | 20 | 30 | 40 | 50 | 60 | 70 | 80 | 90 | 100 |
| --- | --- | --- | --- | --- | --- | --- | --- | --- | --- | --- | --- |

| "The information on speed was useful" | 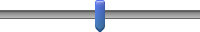 |
| --- | --- |
| "The information on speed was easy to understand" | 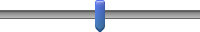 |
| "I would like to have this kind of information available in my own car" | 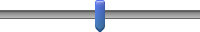 |

Display This Question:

If (filled by experimenter) was this the control condition? = no

At_t2_distance You were presented with information on the **distance to the car ahead**during this ride. What is your opinion regarding the following statements, from 0 "not at all" to 100 "very much"?

|  | 0 | 10 | 20 | 30 | 40 | 50 | 60 | 70 | 80 | 90 | 100 |
| --- | --- | --- | --- | --- | --- | --- | --- | --- | --- | --- | --- |

| "The information on distance was useful" | 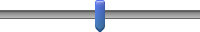 |
| --- | --- |
| "The information on distance was easy to understand" | 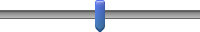 |
| "I would like to have this kind of information available in my own car" | 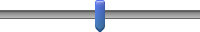 |

End of Block: MW and SA / after ride 2

Start of Block: info2

info4 This is the end of this section of the questionnaire. 

You can give back the laptop to the experimenter and we will resume with the driving part. 

End of Block: info2

Start of Block: MW and SA / after ride 3

condition_t3 Condition (filled by experimenter)

________________________________________________________________

condition_t3_control (filled by experimenter) was this the control condition?

- yes
- no

| Page Break |  |
| --- | --- |

instruction_t3 Based on the scale presented below, how would you rate your mental effort during this ride?

MW_pic_t3

| 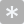 |
| --- |

MW_t3 Response between 0 and 150

________________________________________________________________

| Page Break |  |
| --- | --- |

SA_t3_q1 How much were you concentrating on the road situations? Were you concentrating a lot (High) or a little (Low)?

|  | 1 | 2 | 3 | 4 | 5 | 6 | 7 |
| --- | --- | --- | --- | --- | --- | --- | --- |

| 1 is Low, 7 is High |  |
| --- | --- |

SA_t3_q2 How much was your attention divided during the drive? Were you concentrating on many aspects of the road situations (High) or focused on only one (Low)?

|  | 1 | 2 | 3 | 4 | 5 | 6 | 7 |
| --- | --- | --- | --- | --- | --- | --- | --- |

| 1 is Low, 7 is High |  |
| --- | --- |

SA_t3_q3 How much mental capacity did you have to spare during the drive? Did you have enough capacity to be able to attend to another task (High) or not (Low)?

|  | 1 | 2 | 3 | 4 | 5 | 6 | 7 |
| --- | --- | --- | --- | --- | --- | --- | --- |

| 1 is Low, 7 is High |  |
| --- | --- |

| Page Break |  |
| --- | --- |

Display This Question:

If (filled by experimenter) was this the control condition? = no

At_t3_speed You were presented with information on your **speed** during this ride. What is your opinion regarding the following statements, from 0 "not at all" to 100 "very much"?

|  | 0 | 10 | 20 | 30 | 40 | 50 | 60 | 70 | 80 | 90 | 100 |
| --- | --- | --- | --- | --- | --- | --- | --- | --- | --- | --- | --- |

| "The information on speed was useful" | 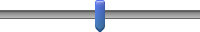 |
| --- | --- |
| "The information on speed was easy to understand" | 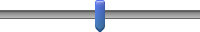 |
| "I would like to have this kind of information available in my own car" | 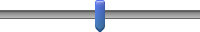 |

Display This Question:

If (filled by experimenter) was this the control condition? = no

At_t3_distance You were presented with information on the **distance to the car ahead**during this ride. What is your opinion regarding the following statements, from 0 "not at all" to 100 "very much"?

|  | 0 | 10 | 20 | 30 | 40 | 50 | 60 | 70 | 80 | 90 | 100 |
| --- | --- | --- | --- | --- | --- | --- | --- | --- | --- | --- | --- |

| "The information on distance was useful" | 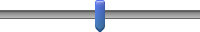 |
| --- | --- |
| "The information on distance was easy to understand" | 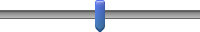 |
| "I would like to have this kind of information available in my own car" | 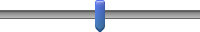 |

End of Block: MW and SA / after ride 3

Start of Block: info3

info5 This is the end of this section of the questionnaire. 

You can give back the laptop to the experimenter and we will resume with the driving part. 

End of Block: info3

Start of Block: MW and SA / after ride 4

condition_t4 Condition (filled by experimenter)

________________________________________________________________

condition_t4_control (filled by experimenter) was this the control condition?

- yes
- no

| Page Break |  |
| --- | --- |

instr_t4 Based on the scale presented below, how would you rate your mental effort during this ride?

MW_pic_t4

| 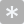 |
| --- |

MW_t4 Response between 0 and 150

________________________________________________________________

| Page Break |  |
| --- | --- |

SA_t4_q1 How much were you concentrating on the road situations? Were you concentrating a lot (High) or a little (Low)?

|  | 1 | 2 | 3 | 4 | 5 | 6 | 7 |
| --- | --- | --- | --- | --- | --- | --- | --- |

| 1 is Low, 7 is High |  |
| --- | --- |

SA_t4_q2 How much was your attention divided during the drive? Were you concentrating on many aspects of the road situations (High) or focused on only one (Low)?

|  | 1 | 2 | 3 | 4 | 5 | 6 | 7 |
| --- | --- | --- | --- | --- | --- | --- | --- |

| 1 is Low, 7 is High |  |
| --- | --- |

SA_t4_q3 How much mental capacity did you have to spare during the drive? Did you have enough capacity to be able to attend to another task (High) or not (Low)?

|  | 1 | 2 | 3 | 4 | 5 | 6 | 7 |
| --- | --- | --- | --- | --- | --- | --- | --- |

| 1 is Low, 7 is High |  |
| --- | --- |

| Page Break |  |
| --- | --- |

Display This Question:

If (filled by experimenter) was this the control condition? = no

At_t4_speed You were presented with information on your **speed** during this ride. What is your opinion regarding the following statements, from 0 "not at all" to 100 "very much"?

|  | 0 | 10 | 20 | 30 | 40 | 50 | 60 | 70 | 80 | 90 | 100 |
| --- | --- | --- | --- | --- | --- | --- | --- | --- | --- | --- | --- |

| "The information on speed was useful" | 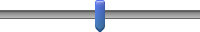 |
| --- | --- |
| "The information on speed was easy to understand" | 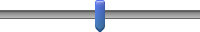 |
| "I would like to have this kind of information available in my own car" | 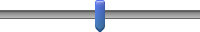 |

Display This Question:

If (filled by experimenter) was this the control condition? = no

At_t4_distance You were presented with information on the **distance to the car ahead**during this ride. What is your opinion regarding the following statements, from 0 "not at all" to 100 "very much"?

|  | 0 | 10 | 20 | 30 | 40 | 50 | 60 | 70 | 80 | 90 | 100 |
| --- | --- | --- | --- | --- | --- | --- | --- | --- | --- | --- | --- |

| "The information on distance was useful" | 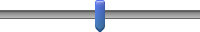 |
| --- | --- |
| "The information on distance was easy to understand" | 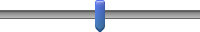 |
| "I would like to have this kind of information available in my own car" | 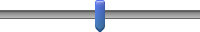 |

End of Block: MW and SA / after ride 4

Start of Block: info 4

info6 This is the end of this section of the questionnaire. 

You can give back the laptop to the experimenter and we will resume with the driving part. 

End of Block: info 4

Start of Block: MW and SA / after ride 5

condition_t5 Condition (filled by experimenter)

________________________________________________________________

condition_t5_control (filled by experimenter) was this the control condition?

- yes
- no

| Page Break |  |
| --- | --- |

instr_t5 Based on the scale presented below, how would you rate your mental effort during this ride?

MW_pic_t5

| 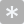 |
| --- |

MW_t5 Response between 0 and 150

________________________________________________________________

| Page Break |  |
| --- | --- |

SA_t5_q1 How much were you concentrating on the road situations? Were you concentrating a lot (High) or a little (Low)?

|  | 1 | 2 | 3 | 4 | 5 | 6 | 7 |
| --- | --- | --- | --- | --- | --- | --- | --- |

| 1 is Low, 7 is High |  |
| --- | --- |

SA_t5_q2 How much was your attention divided during the drive? Were you concentrating on many aspects of the road situations (High) or focused on only one (Low)?

|  | 1 | 2 | 3 | 4 | 5 | 6 | 7 |
| --- | --- | --- | --- | --- | --- | --- | --- |

| 1 is Low, 7 is High |  |
| --- | --- |

SA_t5_q3 How much mental capacity did you have to spare during the drive? Did you have enough capacity to be able to attend to another task (High) or not (Low)?

|  | 1 | 2 | 3 | 4 | 5 | 6 | 7 |
| --- | --- | --- | --- | --- | --- | --- | --- |

| 1 is Low, 7 is High |  |
| --- | --- |

| Page Break |  |
| --- | --- |

Display This Question:

If (filled by experimenter) was this the control condition? = no

At_t5_speed You were presented with information on your **speed** during this ride. What is your opinion regarding the following statements, from 0 "not at all" to 100 "very much"?

|  | 0 | 10 | 20 | 30 | 40 | 50 | 60 | 70 | 80 | 90 | 100 |
| --- | --- | --- | --- | --- | --- | --- | --- | --- | --- | --- | --- |

| "The information on speed was useful" | 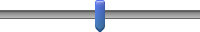 |
| --- | --- |
| "The information on speed was easy to understand" | 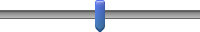 |
| "I would like to have this kind of information available in my own car" | 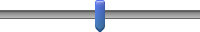 |

Display This Question:

If (filled by experimenter) was this the control condition? = no

At_t5_distance You were presented with information on the **distance to the car ahead**during this ride. What is your opinion regarding the following statements, from 0 "not at all" to 100 "very much"?

|  | 0 | 10 | 20 | 30 | 40 | 50 | 60 | 70 | 80 | 90 | 100 |
| --- | --- | --- | --- | --- | --- | --- | --- | --- | --- | --- | --- |

| "The information on distance was useful" | 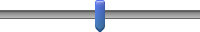 |
| --- | --- |
| "The information on distance was easy to understand" | 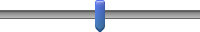 |
| "I would like to have this kind of information available in my own car" | 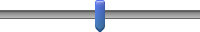 |

End of Block: MW and SA / after ride 5

Start of Block: info 5

last_info This was the last ride! 

The following questions cover the entirety of the experiment, including all different rides. This will be the last section of this questionnaire, and should take no longer than ten minutes. 

End of Block: info 5

Start of Block: OPINIONS AND ATTITUDES (after rides)

enjoyed_feedback What is your opinion regarding the statement "I enjoyed receiving feedback during my driving"?

- Strongly disagree
- Disagree
- Somewhat disagree
- Neither agree nor disagree
- Somewhat agree
- Agree
- Strongly agree

difficulty_speed_ass What is your opinion regarding the statement "I sometimes have difficulties assessing the speed I am driving at"?

- Strongly disagree
- Disagree
- Somewhat disagree
- Neither agree nor disagree
- Somewhat agree
- Agree
- Strongly agree

difficulty_dist_ass What is your opinion regarding the statement "I sometimes have difficulties assessing the distance between my car and the car in front of me"?

- Strongly disagree
- Disagree
- Somewhat disagree
- Neither agree nor disagree
- Somewhat agree
- Agree
- Strongly agree

| Page Break |  |
| --- | --- |

instruction_ This set of questions concerns the feedback on speed

rating_speed_useful You were presented three different forms of feedback regarding your **speed**. Please rate them, **based on their usefulness** (with 0 being not useful at all and 100 being very useful).

|  | 0 | 10 | 20 | 30 | 40 | 50 | 60 | 70 | 80 | 90 | 100 |
| --- | --- | --- | --- | --- | --- | --- | --- | --- | --- | --- | --- |

| Condition 1 | 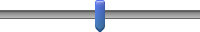 |
| --- | --- |
| Condition 2 | 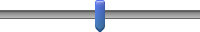 |
| Condition 3 | 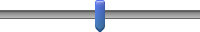 |

intent_use_speed Assuming that all three options of feedback on speed are available in your car, and that you have the choice to use them or not. Please indicate **the probability that you would use** the following forms of feedback (with 0 being no chance of using the feedback and 100 being definite intention to use the feedback).

|  | 0 | 10 | 20 | 30 | 40 | 50 | 60 | 70 | 80 | 90 | 100 |
| --- | --- | --- | --- | --- | --- | --- | --- | --- | --- | --- | --- |

| Condition 1 | 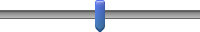 |
| --- | --- |
| Condition 2 | 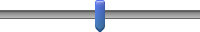 |
| Condition 3 | 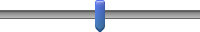 |

ranking_speed You were presented three different forms of feedback regarding your speed. Please **rank the feedback based on your preference** (1 being your preferred feedback and 3 your least preferred).

______

______

______

| Page Break |  |
| --- | --- |

instruction_2 This set of questions concerns the feedback on distance to the vehicle ahead

rating_dist_useful You were presented four different forms of feedback regarding **the distance to the vehicle ahead of you**. Please rate them, based on their **usefulness** (with 0 being not useful at all and 100 being very useful).

|  | 0 | 10 | 20 | 30 | 40 | 50 | 60 | 70 | 80 | 90 | 100 |
| --- | --- | --- | --- | --- | --- | --- | --- | --- | --- | --- | --- |

| Condition 1 | 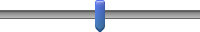 |
| --- | --- |
| Condition 2 | 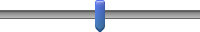 |
| Condition 3 | 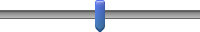 |
| Condition 4 | 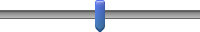 |

intent_use_distance Assuming that all four options of feedback on distance to the vehicle ahead of you are available in your car, and that you have the choice to use them or not. Please indicate the **probability that you would use** the following forms of feedback (with 0 being no chance of using the feedback and 100 being definite intention to use the feedback).

|  | 0 | 10 | 20 | 30 | 40 | 50 | 60 | 70 | 80 | 90 | 100 |
| --- | --- | --- | --- | --- | --- | --- | --- | --- | --- | --- | --- |

| Condition 1 | 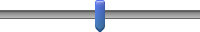 |
| --- | --- |
| Condition 2 | 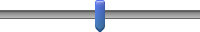 |
| Condition 3 | 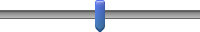 |
| Condition 4 | 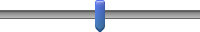 |

ranking_distance You were presented four different forms of feedback regarding the distance to the vehicle ahead. Please **rank the feedback based on your preference** (1 being your preferred feedback and 3 your least preferred).

______

______

______

______

End of Block: OPINIONS AND ATTITUDES (after rides)

Start of Block: MW and SA overall (after all the rides)

mw_afterrides Please rate the **mental effort** you exerted during the rides of the following conditions, with 0 being no effort at all and 100 being the biggest effort.

|  | 0 | 10 | 20 | 30 | 40 | 50 | 60 | 70 | 80 | 90 | 100 |
| --- | --- | --- | --- | --- | --- | --- | --- | --- | --- | --- | --- |

| With no feedback | 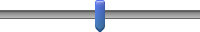 |
| --- | --- |
| Condition 1 | 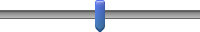 |
| Condition 2 | 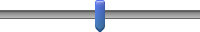 |
| Condition 3 | 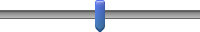 |
| Condition 4 | 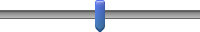 |

sa_afterrides Please rate your **awareness** during the rides of the following conditions (how aware of your surroundings you were), with 0 being not aware at all and 100 being the most aware.

|  | 0 | 10 | 20 | 30 | 40 | 50 | 60 | 70 | 80 | 90 | 100 |
| --- | --- | --- | --- | --- | --- | --- | --- | --- | --- | --- | --- |

| With no feedback | 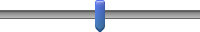 |
| --- | --- |
| Condition 1 | 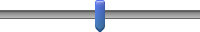 |
| Condition 2 | 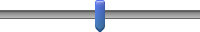 |
| Condition 3 | 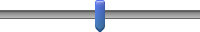 |
| Condition 4 | 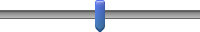 |

End of Block: MW and SA overall (after all the rides)

Start of Block: end

Q116 This is the end of the study! 

 Thank you again very much for participating. If you have any remarks or questions, you can address them to the experimenter. 


 **Do you want to participate in the lottery to have a chance to win 25 euros? Then register on the next page!**

End of Block: end

Start of Block: lottery registration

Do you want to parti Do you want to participate in the lottery to have a chance to win 25 euros?

- Yes
- No

| 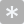 |
| --- |

Q118 In case you win, we will contact you via email to collect your information (such as your IBAN). What is your email address?

________________________________________________________________

Q119 The draw will be made at the very end of the data collection, at the end of the month of January. The three winners will be contacted then.

End of Block: lottery registration
